# Genotype-Phenotype correlations in Joubert Syndrome in the Era of Next Generation Sequencing

**DOI:** 10.1186/2046-2530-4-S1-P8

**Published:** 2015-07-13

**Authors:** R Bachmann-Gagescu, J Dempsey, IG Phelps, C Isabella, D O'Day, B O'Roak, J Shendure, I Glass, D Doherty

**Affiliations:** 1 Institute for Molecular Life Sciences and Institute for Medical Genetics, University of Zurich, Zurich, Switzerland; 2 Pediatrics, University of Washington, Seattle, WA, USA; 3 Genome Sciences, University of Washington, Seattle, WA, USA

## Objective

To provide extensive genotype-phenotype correlations for Joubert syndrome (JS), a ciliopathy characterized by a distinctive hindbrain malformation ("the molar tooth sign"), ataxia and cognitive dysfunction.

## Methods

Phenotypic data was collected from the University of Washington JS cohort and all known JS genes were sequenced in 429 individuals (364 families) using the MIPS capture technique and next-generation sequencing.

## Results

Core JS diagnostic features (hypotonia, ataxia, cognitive dysfunction, oculo-motor apraxia) were present in >80% of individuals, while abnormal breathing pattern was reported in 60%. Frequently associated features included retinal dystrophy (31.4%), renal disease (20.9%), coloboma (17.7%), polydactyly (15.3%), liver fibrosis (15.2%) and encephalocele (8%). Liver fibrosis and coloboma were strongly associated with each other (Odds Ratio 7.0, 95% Confidence Interval = 3.0-13.2), while retinal dystrophy and renal disease were weakly associated (O.R. 2.2, 95%C.I. = 1.7-5.6). Additional clinical features included other brain abnormalities (n = 73), seizures (n = 49), cleft palate (n = 16), hearing loss (n = 14) and psychiatric problems (n = 45). The genetic cause was identified in 60% of families, with 5 genes accounting for the majority of patients (*C5ORF42, CEP290, CC2D2A, AHI1*, *TMEM67)*. Bi-allelic causal mutations in *B9D2 *and *C2CD3 *were identified in 2 families each. Bi-allelic mutations in 2 different genes were identified in 4 families and heterozygous mutations (in addition to the causal mutation) were present in 62 individuals. Significant (p<0.0001) genotype-phenotype correlations were observed: *CEP290 *with renal disease and retinal dystrophy; *TMEM67 *with liver fibrosis and coloboma.

## Conclusion

This study provides a comprehensive description of the phenotypic spectrum, genetic makeup and genotype-phenotype correlations of a large JS cohort.

